# Grading cervical neural foraminal stenosis via 3-T MR nerve/bone fusion imaging compared with T2-weighted imaging

**DOI:** 10.1186/s13244-025-02094-3

**Published:** 2025-10-16

**Authors:** Dongmei Jiang, Shuyi Lei, Junhuan Hong, Xiang Lin, Ruiquan Chen, Dejun She, Dairong Cao

**Affiliations:** 1 Department of Radiology, First Affiliated Hospital of Fujian Medical University, Fuzhou, P.R. China; 2 Department of Radiology, National Regional Medical Center, Binhai Campus of the First Affiliated Hospital, Fujian Medical University, Fuzhou, P.R. China; 3 Key Laboratory of Radiation Biology of Fujian Higher Education Institutions, The First Affiliated Hospital, Fujian Medical University, Fuzhou, P.R. China; 4 Department of Radiology, Fujian Key Laboratory of Precision Medicine for Cancer, The First Affiliated Hospital, Fujian Medical University, Fuzhou, P.R. China

**Keywords:** Cervical radiculopathy, Cervical neural foraminal stenosis, Grade of stenosis, MRI nerve/bone fusion

## Abstract

**Objectives:**

To investigate the value of 3-T MR nerve/bone fusion imaging in grading cervical neural foraminal stenosis (CNFS).

**Materials and methods:**

Fifty-eight healthy participants and 23 patients with suspected cervical radiculopathy were prospectively enrolled. MR nerve and bone sequences were 3D-T2-weighted fast field echo (3D-T2-FFE) and fast field echo resembling a CT using restricted echo-spacing (FRACTURE), respectively. The agreements of overall image quality, image artifacts, the width of cervical neural foramen (WCNF), and the width of extraforaminal nerve root (WENR) were assessed on 3D-T2-FFE/FRACTURE fusion images in healthy participants. The detection rate, visibility score of extraforaminal nerve root (ENR), and the CNFS grade were compared for patients between the 3D-T2-FFE/FRACTURE fusion image and the T2-weighted images (T2WI). The correlation between CNFS grade and the neck disability index (NDI) and numerical pain scale (NPS) was assessed.

**Results:**

The agreements were moderate to good for overall image quality and image artifacts (*κ* = 0.614–0.867), and good to excellent for WCNF and WENR (ICC = 0.755–0.931). The detection rate of ENR on 3D-T2-FFE/FRACTURE fusion (184/184, 100%) was higher than that on T2WI (116/184, 63.04%). The agreements for CNFS grade were substantial to nearly perfect on 3D-T2-FFE/FRACTURE fusion (*κ* = 0.774–0.837), and moderate on T2WI (*κ* = 0.436–0.636). The CNFS grade on 3D-T2-FFE/FRACTURE fusion was moderately correlated with NDI (ρ = 0.49)/NPS (ρ = 0.55), while there was no correlation between T2WI and NDI/NPS.

**Conclusion:**

Compared with T2WI, 3D-T2-FFE/FRACTURE fusion provides a more reliable and reproducible evaluation of the severity of CNFS. The CNFS grade based on 3D-T2-FFE/FRACTURE fusion is associated with clinical symptoms.

**Critical relevance statement:**

The use of 3-T MR nerve/bone fusion imaging in clinical practice may facilitate a one-stop-shop, radiation-free, and more precise approach to comprehensively evaluate CNFS.

**Key Points:**

- Grading cervical foraminal stenosis is relatively difficult in clinical practice.
- MR nerve/bone fusion grading of cervical foraminal stenosis is more reliable.
- MR nerve/bone fusion grading of cervical foraminal stenosis correlates with symptoms.

**Graphical Abstract:**

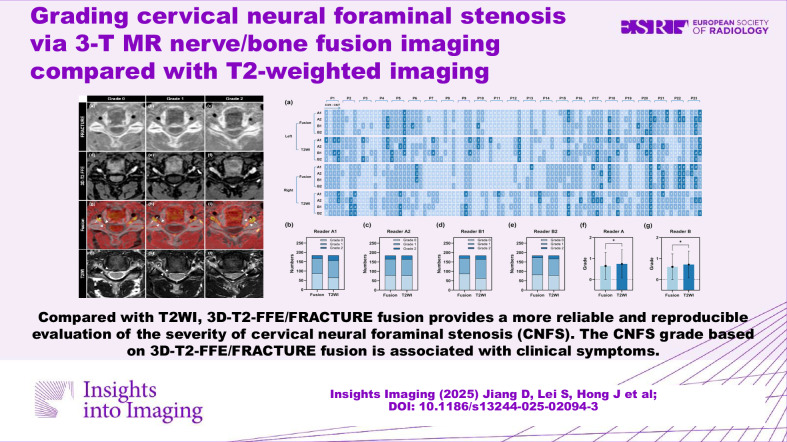

## Introduction

Cervical neural foraminal stenosis (CNFS) is mostly caused by lateral disc herniation and bony hypertrophy [1]. It was reported that about 10–25% of the adult population has CNFS [2]. CNFS may cause cervical radiculopathy when the cervical nerve root is compressed [3, 4], resulting in neck and arm pain [5]. The precise evaluation of CNFS is important for determining appropriate treatment methods and predicting prognosis [6, 7]. Therefore, it is significant to choose an appropriate imaging modality to evaluate CNFS.

Magnetic resonance imaging (MRI) is the most important modality for evaluating CNFS, providing detailed visualization of the soft tissue [8, 9]. Axial T2-weighted images (T2WI) have been used for grading CNFS by Kim et al [10]. However, it is relatively difficult in clinical practice. According to this grading system, the narrowest width of the cervical neural foramen (WCNF) and the width of the extraforaminal nerve root (WENR) were used to grade CNFS. On the one hand, the extraforaminal nerve root (ENR) and the neural foramen structures are small, bringing the difficulty in comparing the WENR and WCNF on T2WI. On the other hand, conventional T2WI is inferior in its ability to outline bony structures [11], resulting in inaccurate measurement for WCNF. Therefore, if an imaging modality can visualize both bony cervical neural foramina and ENR, it may facilitate a more precise grade of CNFS.

It was reported that a 3D CT/MRI fusion model has been used for evaluating cervical nerve root and cervical bony spur together [12]. However, this fusion technique requires two different imaging modalities, which could involve cumbersome and error-prone steps. Furthermore, 3D CT/MRI fusion needed extra CT examination containing radiation exposure risks, which may be harmful for the thyroid and not suitable for pregnant women. Thus, 3D CT/MRI fusion is limited in clinical practice.

With the development of new MRI technology, several novel sequences have been used to assess CNFS. Magnetic resonance neurography (MRN) can directly visualize nerves by selectively highlighting water within the endoneurium [13, 14]. It has been shown that MRN can evaluate compression of perineural nerves in patients with degenerative disc diseases [15, 16]. CT-like MRI technique has been used to evaluate osseous CNFS, such as zero echo time (ZTE) sequence [17], susceptibility-weighted imaging (SWI) [18], and fast-field-echo resembling a CT scan using restricted echo-spacing (FRACTURE) [19, 20]. According to previous studies, 3-T MR nerve/bone fusion imaging can effectively assess the relationship between impacted mandibular third molar and inferior alveolar nerve [21] or lingual nerve [22]. However, the value of 3-T MR nerve/bone fusion imaging in evaluating CNFS remains unknown.

We hypothesize that 3-T MR nerve/bone fusion imaging can clearly show cervical nerve root and cervical neural foramen simultaneously. The aim of this study was to investigate the value of 3-T MR nerve/bone fusion imaging in evaluating CNFS compared with T2WI and to assess the correlation between the severity of CNFS and clinical symptoms.

## Materials and methods

### Study design and participants

This prospective study received approval from our institutional review board, and all participants provided written informed consent (Approval number: MRCTA, ECFAH of FMU [2023] 434).

This study initially included 60 healthy participants between July 1, 2023, and November 28, 2023. Inclusion criteria were: (1) no cervical spondylosis, inflammation, tumor, trauma, or surgery; (2) no contraindications of MRI examination. Exclusion criteria were: (1) failure to complete all MRI sequences; (2) significant image artifacts.

Besides, this study initially included 45 consecutive patients with suspected cervical radiculopathy who visited our institution because of neck pain, radiating pain or numbness in the upper extremities, and weakness of the hand or finger, and underwent CT of the cervical spine between January 12, 2024, and July 23, 2024. Exclusion criteria were: (1) failure to complete all MRI sequences, (2) significant image artifacts, (3) central disc herniation, (4) history of cervical surgery or trauma, and (5) contraindications of MRI examination.

### Image acquisition

MRI examinations were performed using a 3-T whole-body MR scanner (Philips Ingenia, Best, The Netherlands) with a 16-channel head-neck coil. No contrast agent was administered for any of the imaging sequences used in this study.

MR nerve and bone sequences performed in this study were 3D-T2-weighted fast field echo (3D-T2-FFE) and fast field echo resembling a CT using restricted echo-spacing (FRACTURE), respectively. 3D-T2-FFE can visualize the cervical nerve root by selectively highlighting water within the endoneurium. FRACTURE employs multiple echoes with a constant echo spacing and post-processing subtraction to generate a CT-like image [23].

For healthy participants, coronal 3D-T2-FFE and FRACTURE examination were performed. Each healthy participant was examined twice on 2 separate days, 7 days apart. For patients, conventional cervical MRI, 3D-T2-FFE, and FRACTURE were performed. Detailed MRI protocol parameters were provided in Table S1.

### Image post-processing

Image post-processing was performed by two radiologists with different clinical experience (7 years and 12 years) together using the Philips MR workstation (IntelliSpace Portal, ISP), including the generation of CT-like images and fusion of 3D-T2-FFE and FRATURE sequences.

### Generation of CT-like images

**Fig. 1:**
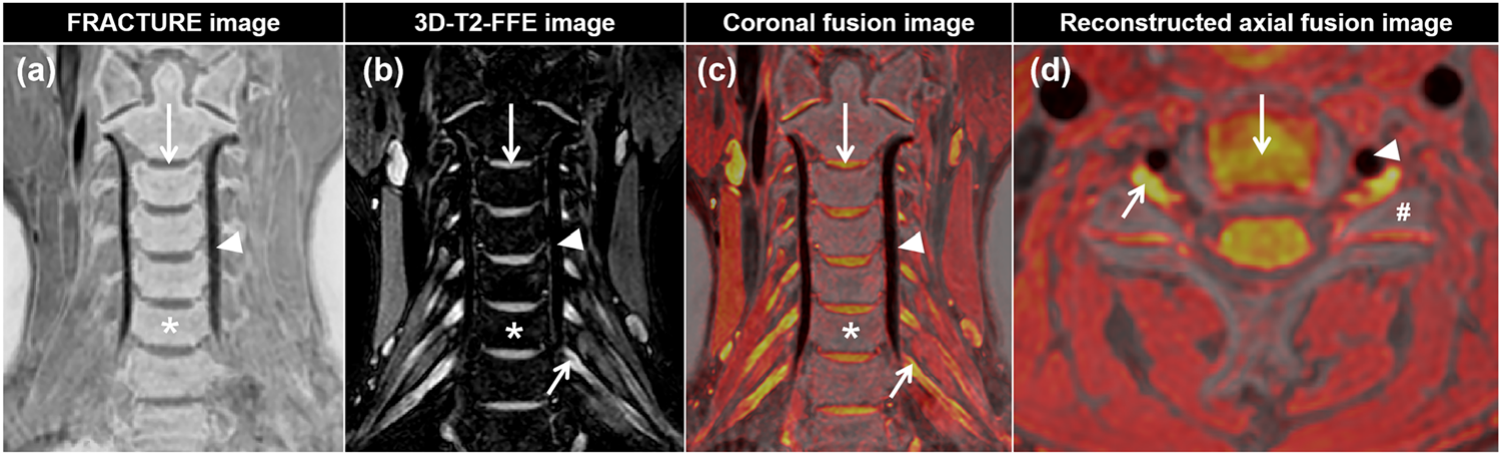
Visualization of the intervertebral disc (long arrow), cervical nerve root (short arrow), vertebral artery (arrowhead), vertebral body (*), and superior articular process (#) on FRACTURE (**a**), 3D-T2-FFE (**b**), and 3D-T2-FFE/FRACTURE fusion images (**c**, **d**)

**Fig. 2:**
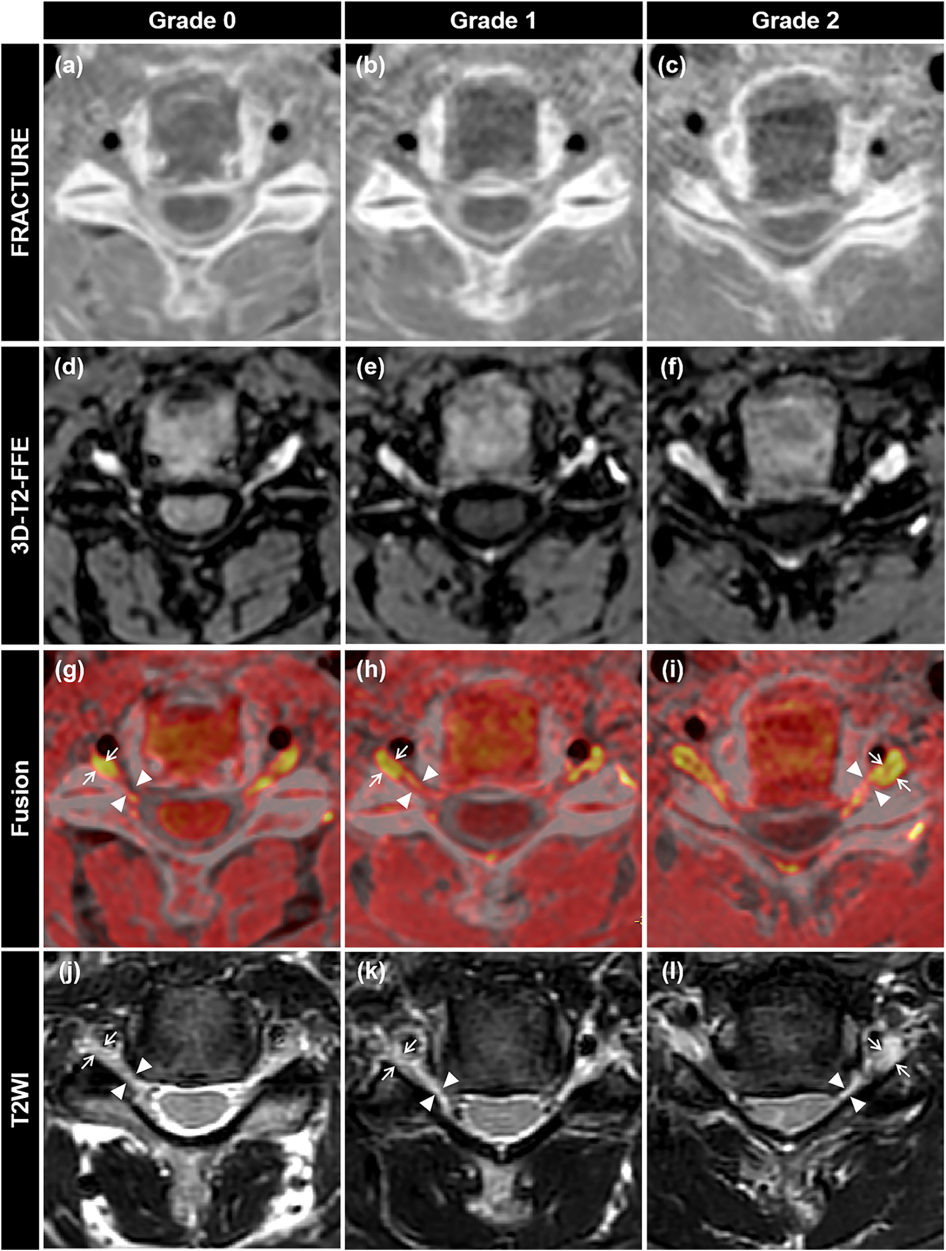
Illustrative diagrams of FRACTURE images (**a**–**c**), 3D-T2-FFE images (**d**–**f**), and CNFS grades on axial 3D-T2-FFE/FRACTURE fusion (**g**–**i**) and T2WI (**j**–**l**). 3D-T2-FFE/FRACTURE fusion images (**g**–**i**) were obtained by fusing FRACTURE images (**a**–**c**) and 3D-T2-FFE images (**d**–**f**). **g**, **j** Grade 0, WCNF (arrowheads) greater than WENR (arrows). **h**, **k** Grade 1, WCNF (arrowheads) greater than 50% of WENR (arrows). **i**, **l** Grade 2, WCNF (arrowheads), same or less than 50% of WENR (arrows)

The magnitudes of 6 echoes were summed [23] using the “MR Echo Accumulation” module first. Then the sixth echo was subtracted from the summed images [23] using the “MR Subtraction” module to invert grayscale and enhance bone contrast. The generated CT-like images are shown in Figs. 1a and 2a–c.

### Fusion of 3D-T2-FFE and FRACTURE images

Image fusion was carried out in the “Multi-Modality Viewer” module. FRACTURE images were set as the primary images, and 3D-T2-FFE images as the floating images. Automatic and manual registration were performed to ensure the precise alignment of the 2 sets of images across axial, coronal, and sagittal planes. Automatic registration was performed first. Then we observed layer by layer to confirm whether the intervertebral disc, vertebral artery, muscle-fat interfaces, spinal cord, and bone cortex matched on the 2 sets of images. We also observed using reconstructed axial and sagittal images. If these anatomical structures were judged to match well on the 2 sets of images by the two radiologists, the image fusion process was completed. Otherwise, manual registration was performed through fine image rotation and translation until a satisfactory fusion image was obtained, averaging about 1 min per case. In this study, 52 participants (37 healthy participants and 15 patients) achieved satisfactory fusion with automatic registration, while 29 participants (21 healthy participants and 8 patients) required manual registration. In the fusion images (Fig. 1c, d), nerve root, intervertebral disc, and spinal cord were displayed in yellow, cortical bone in gray, and muscles in red.

### Image assessment

Image assessments were performed using the Philips workstation (IntelliSpace Portal, ISP). All images were assessed on axial T2WI and 3D-T2-FFE/FRACTURE fusion images.

### Reliability and reproducibility of 3D-T2-FFE/FRACTURE fusion

The data of 58 healthy participants were used to assess the reliability and reproducibility of 3D-T2-FFE/FRACTURE fusion images. A total of 580 foramina and corresponding nerve roots of 58 healthy participants from bilateral C2/3–C6/7 were qualitatively and quantitatively evaluated. Qualitative analyses included evaluations of overall image quality and image artifacts using 4-point Likert scales (Table 1). Quantitative analyses included measurements of WCNF and WENR.

**Table 1:** Likert scale criteria for the assessment of overall image quality, artifacts, ENR visibility, and confidence of grading

Score		Criterion description
Overall image quality^a^		
1	Poor	Cervical nerve roots and neural foramina are blurred and unrecognizable
2	Fair	Cervical nerve roots and neural foramina are blurred but remain identifiable
3	Good	Cervical nerve roots and neural foramina are visible with a heterogeneous signal
4	Excellent	Cervical nerve roots and neural foramina are sharply delineated with a homogeneous signal
Image artifacts^a^		
0	Severe	Anatomical details are blurred and unrecognizable due to massive artifacts
1	Moderate	Anatomical details are blurred but remain identifiable due to moderate artifact
2	Mild	Anatomical details are visible with minor artifact
3	None	Anatomical details are sharply delineated without artifacts
Visibility of the ENR^b^		
1	None	ENR is not visualized
2	Poor	ENR is blurred and poorly discernible
3	Fair	ENR is blurred but remains identifiable
4	Good	ENR is clearly visible with a heterogeneous signal
5	Excellent	ENR is sharply delineated with a homogeneous signal
Confidence of grading^c^		
1	Uncertain	Uncertain of neural foramina and ENR clarity, grading is challenging
2	Fair	Moderately confident in neural foramina and ENR clarity, needing additional confirmation
3	Certain	Highly confident in neural foramina and ENR clarity, allowing for easy grading

*ENR* extraforaminal nerve root

^a^ Qualitative analysis of 3D-T2-FFE/FRACTURE fusion images using a 4-point Likert scale in 58 healthy participants

^b^ Assessment of ENR visibility on 3D-T2-FFE/FRACTURE fusion images and T2WI using a 5-point Likert scale in 23 patients

^c^ Confidence level when grading CNFS using the 3D-T2-FFE/FRACTURE fusion images vs T2WI in 23 patients

These images were evaluated in a randomized order by two readers (Reader A, a second-year graduate student in medical imaging, and Reader B, a musculoskeletal radiologist with 13 years of clinical experience). To assess the intra-reader and test–retest agreement, each reader interpreted the images twice at an interval of 4 weeks to minimize memory bias. The design of the assessment for the reliability and reproducibility of the 3D-T2-FFE/FRACTURE fusion technique is detailed in Fig. S1.

### Visibility of the ENR

The visibility of ENR from bilateral C3/4 to C6/7 (184 foramina) was evaluated by readers A and B using data from 23 patients. The number of cervical neural foramina (*N*_CNF_ = 184) and identified ENR (*N*_ENR_) were recorded. And the detection rate of ENR was calculated by Eq. (1). Besides, the visibility score of ENR was evaluated using a 5-point Likert scale (Table 1).

1 $${{{\rm{ENR}}}}\; {{{\rm{detection}}}}\; {{{\rm{rate}}}}=\frac{{{{N}}}_{{{\rm{ENR}}}}}{{{{N}}}_{{{\rm{CNF}}}}}\times 100 \%$$

### Grade of CNFS

The grade of CNFS from bilateral C3/4 to C6/7 (184 foramina) was evaluated by reader A and reader B independently. Both readers assessed the grade of CNFS twice at the narrowest point according to the grading system suggested by Kim et al [10]. To assess the intra-reader agreement, each reader interpreted the images twice at an interval of 4 weeks to minimize memory bias. Grading criteria for CNFS were as follows [10]: grade 0, normal = the narrowest WCNF greater than WENR at the level of the anterior margin of the superior articular process (Fig. 2g, j); grade 1, non-severe = the narrowest WCNF is 51–100% of WENR at the level of the anterior margin of the superior articular process (Fig. 2h, k); grade 2, severe = the narrowest WCNF is the same as or less than 50% of WENR at the level of the anterior margin of the superior articular process (Fig. 2i, l). In cases of an unclear ipsilateral ENR on axial T2WI, the width of the contralateral ENR at the level of the superior articular process or the width between the posterior margin of the vertebral artery and the anterior margin of the superior articular process was alternatively used [10]. We recorded the time it took when grading CNFS using 3D-T2-FFE/FRACTURE fusion images and T2WI. Besides, we also applied a 3-point Likert scale (Table 1) to compare how confident the two readers felt when grading CNFS using the 3D-T2-FFE/FRACTURE fusion images vs T2WI.

### Clinical correlation between the grade of CNFS and NDI/NPS

In patients with cervical radiculopathy, the neck disability index (NDI) and numerical pain scale (NPS) are the most frequently used patient-reported outcome measures, focusing on patient physical function and pain. When patients came to our institution for MRI examination, they all underwent clinical scoring of the neck and arms using NDI and NPS. The NDI questionnaire has ten items concerning pain and activities of daily living [24] (Appendix S1). The total NDI score is calculated by Eq. (2). The NPS uses a discontinuous and segmented scale [25]. The patient was asked to mark on the scale to rate their pain, and then the number was recorded [26] (Appendix S2 and Fig. S2). The relationship between CNFS grade and NDI/NPS was analyzed.

2 $${{{\rm{Total}}}}\; {{{\rm{NDI}}}}\; {{{\rm{score}}}}=\frac{{{{\rm{total }}}}\;{{{\rm{scores}}}} \;{{{\rm{of}}}} \;{{{\rm{items}}}} \;{{{\rm{answered}}}}}{5\times {{{\rm{number}}}}\; {{{\rm{of}}}} \;{{{\rm{items}}}} \;{{{\rm{answered}}}}}\times 100 \%$$

### Structured training protocol for image assessment

Prior to the formal image assessment, both readers (A and B) received a summarized table of Likert scales (Table 1) and an unambiguous written description of the CNFS grading system, accompanied by illustrative diagrams (Fig. 2). This was done to ensure their familiarity with the Likert scales, measurements of WCNF/WENR, and Kim’s grading system. A calibration session was then conducted, in which the two readers independently analyzed five randomly selected patients and five randomly selected healthy participants. Finally, they participated in a consensus meeting to compare their assessments and resolve discrepancies. When assessing the ENR visibility and CNFS grade, disagreements between the first reading of reader A and reader B were resolved through a consensus read, which allowed for further comparison between 3D-T2-FFE/FRACTURE fusion images and T2WI.

### Statistical analysis

Statistical analyses were conducted using MedCalc (Version 22.026), online SPSS analysis software (https://spssau.com/index.html), and IBM SPSS Statistics for Windows (Version 26.0, IBM Corp.). Graphs were created using GraphPad Prism 8 (GraphPad Software Inc.), Origin Pro 2022 (OriginLab Inc.), and Hiplot Pro (https://hiplot.com.cn/). Data normality was assessed using the Shapiro–Wilk test (*n* ≤ 50) and Kolmogorov–Smirnov test (*n* > 50) (*p* > 0.05 indicated normal distribution). Continuous variables with normal distribution were expressed as mean ± standard deviation (SD), while non-normally distributed variables were reported as median (interquartile range, IQR).

Agreements for qualitative metrics were assessed using linear weighted Cohen’s kappa (*κ*) with two-sided 95% confidence interval (CI), and agreements were categorized as poor (*κ* < 0.1), slight (0.1 ≤ *κ* ≤ 0.2), fair (0.2 < *κ* ≤ 0.4), moderate (0.4 < *κ* ≤ 0.6), substantial (0.6 < *κ* ≤ 0.8), or nearly perfect (0.8 < *κ* ≤ 1.0) [27]. Agreements for quantitative measurements were assessed using a two-way random intraclass correlation coefficient (ICC) with two-sided 95% CI and Bland–Altman plots. ICC values were categorized as poor (ICC < 0.5), moderate (0.5 < ICC < 0.75), good (0.75 < ICC < 0.9), or excellent (ICC > 0.9) [28].

Detection rates of ENR were compared using the χ^2^ test. The visibility scores of ENR, confidence scores for grading, and grades of CNFS were compared using the Mann–Whitney *U*-test. An independent samples *T*-test was used to compare the time for CNFS grading between 3D-T2-FFE/FRACTURE fusion images and T2WI. Spearman’s partial correlation analysis was performed to explore the independent correlation between CNFS grade and NDI/NPS scores while controlling for age, sex, body mass index (BMI), and months of disease duration. Correlation coefficients (ρ) were interpreted as follows [29]: ρ = 0.00–0.10, negligible correlation; ρ = 0.10–0.39, weak correlation; ρ = 0.40–0.69, moderate correlation; ρ = 0.70–0.89, strong correlation; ρ = 0.90–1.00, very strong correlation.

## Results

### Demographic and clinical characteristics

Two healthy participants (2/60) were excluded from the study due to significant image artifacts. A total of 22 patients (22/45) were excluded due to failure to complete all MRI sequences (*n* = 2), central disc herniation (*n* = 17), history of cervical surgery (*n* = 2), or trauma (*n* = 1). Finally, a total of 58 healthy participants (29 women, 29 men; mean age, 24.03 ± 3.43 years; age range, 12–34 years) and 23 patients with suspected cervical radiculopathy (12 women, 11 men; mean age, 55.48 ± 12.45 years; age range, 37–83 years) were enrolled in the study. Table 2 summarizes the participants’ demographic and clinical characteristics.

**Table 2:** Demographic and clinical characteristics of all participants

	Patients (*n* = 23)	Healthy participants (*n* = 58)
Demographics		
Age [years, mean ± SD]	55.48 ± 12.45	24.03 ± 3.43
Sex [*n*, female/male]	12/11	29/29
BMI [kg/m^2^, mean ± SD]	24.89 ± 3.74	21.81 ± 2.10
Disease duration [months, median (IQR)]	6 (3, 24)	NA
Medical history		
Hypertension [yes, *n* (%)]	4 (17.39%)	0 (0%)
Other comorbidities^a^ [yes, *n* (%)]	0 (0%)	0 (0%)
Clinical assessment		
NDI [score, mean ± SD]	0.28 ± 0.20	NA
NPS [score, mean ± SD]	3.26 ± 2.34	NA
CNFS grade		
Fusion [grade, median (IQR)]	1 (1, 2)	NA
Grading time^b^ [min, mean ± SD]	6.43 ± 1.05	NA
Grading confidence^b^ [score, median (IQR)]	3 (2, 3)	NA
T2WI [grade, median (IQR)]	1 (1, 2)	NA
Grading time^c^ [min, mean ± SD]	9.97 ± 1.02	NA
Grading confidence^c^ [score, median (IQR)]	2 (1, 2)	NA

*NA* not applicable, *BMI* body mass index, *NDI* neck disability index, *NPS* numerical pain scale, *CNFS* cervical neural foraminal stenosis, *Fusion* 3D-T2-FFE/FRACTURE fusion

^a^ Other comorbidities include diabetes mellitus, stroke, coronary heart disease, hyperlipidemia, smoking, and alcohol consumption. No cases were reported in either group

^b^ Average time and confidence in grading CNFS using 3D-T2-FFE/FRACTURE fusion images

^c^ Average time and confidence in grading CNFS using T2WI

### Reliability and reproducibility of 3D-T2-FFE/FRACTURE fusion

The overall image quality of 3D-T2-FFE/FRACTURE fusion images was excellent with an average score of 4.00 (0.00). No image artifacts were observed on 3D-T2-FFE/FRACTURE fusion images with an average score of 0.00 (0.00).

The intra-reader and inter-reader agreements for overall image quality and image artifacts were good, with *κ* values of 0.846 (95% CI: 0.686–1.006) to 0.867 (95% CI: 0.689–1.045). The test–retest agreements for overall image quality and image artifacts were moderate, with *κ* values of 0.614 (95% CI: 0.338–0.890) to 0.716 (95% CI: 0.418–1.013). The intra-reader and inter-reader agreements for WCNF were excellent, with ICC values of 0.925 (95% CI: 0.891–0.947) to 0.931 (95% CI: 0.911–0.946). The test–retest agreements for WCNF were good, with ICC values of 0.835 (95% CI: 0.797–0.867) to 0.855 (95% CI: 0.820–0.884). The intra-reader, inter-reader, and test–retest agreements for WENR were good, with ICC values of 0.755 (95% CI: 0.690–0.806) to 0.874 (95% CI: 0.843–0.898). The intra-reader and inter-reader agreements for the ratio of WCNF/WENR were good, with ICC values of 0.760 (95% CI: 0.686–0.815) to 0.861 (95% CI: 0.827–0.888) (Table 3).

**Table 3:** Reliability and reproducibility of 3D-T2-FFE/FRACTURE fusion imaging

	Inter-reader agreement		Intra-reader agreement		Test–retest agreement	
	Reader A1_first_ vs Reader B1		Reader A1_first_ vs Reader A1_second_		Reader B1 vs Reader B2	
	Left	Right	Left	Right	Left	Right
WCNF^a^	0.930	0.931	0.930	0.925	0.835	0.855
	[0.913–0.944]	[0.911–0.946]	[0.904–0.948]	[0.891–0.947]	[0.797–0.867]	[0.820–0.884]
WENR^a^	0.860	0.874	0.769	0.755	0.787	0.801
	[0.826–0.888]	[0.843–0.898]	[0.700–0.822]	[0.690–0.806]	[0.738–0.828]	[0.756–0.839]
WCNF/WENR^a^	0.861	0.850	0.804	0.760	0.740	0.776
	[0.827–0.888]	[0.805–0.884]	[0.714–0.816]	[0.686–0.815]	[0.683–0.788]	[0.725–0.819]
Overall image quality^b^	0.846 [0.686–1.006]		0.867 [0.689–1.045]		0.716 [0.418–1.013]	
Image artifacts^b^	0.860 [0.664–1.056]		0.858 [0.696–1.020]		0.614 [0.338–0.890]	

*WCNF* width of the cervical neural foramen, *WENR* width of the extraforaminal nerve root, *Reader A1*_first_ first reading of reader A about MR scan 1, *Reader A1*_second_ second reading of reader A about MR scan 1, *Reader B1* reading of reader B about MR scan 1, *Reader B2* reading of reader B about MR scan 2

^a^ ICC; data were given with a two-sided 95% CI

^b^ Weighted Cohen Kappa Coefficient (*κ*); data were given with two-sided 95% CI

**Fig. 3:**
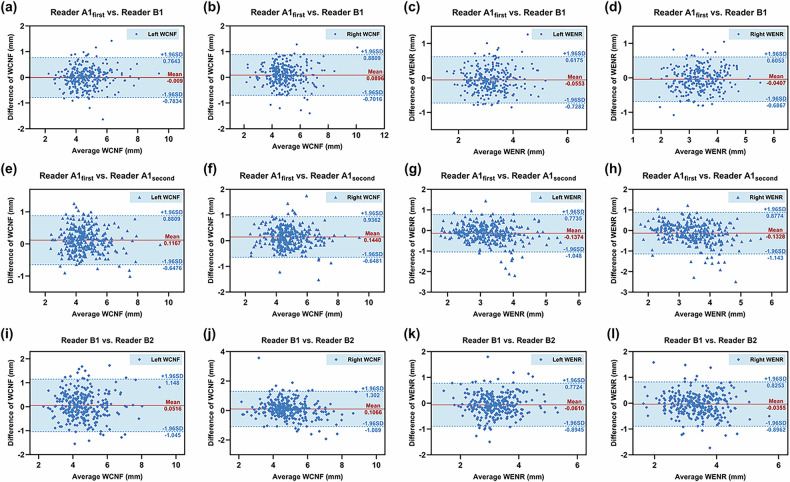
Bland–Altman plots showing inter-reader (**a**–**d**), intra-reader (**e**–**h**), and test–retest (**i**–**l**) agreements of WCNF and WENR. Solid red line: mean differences (bias); dashed blue lines and shaded blue parts: 95% limits of agreement (LoA). Reader A1_first/second_, reader A’s first/second read of MR scan 1; Reader B1/2, reader B’s reads of MR scan 1/2

Bland–Altman analysis demonstrated a random distribution of measurement errors with low bias and relatively narrow inter-reader, intra-reader, and test–retest variation about measurements of WCNF and WENR (Fig. 3).

### Visibility of the ENR

**Fig. 4:**
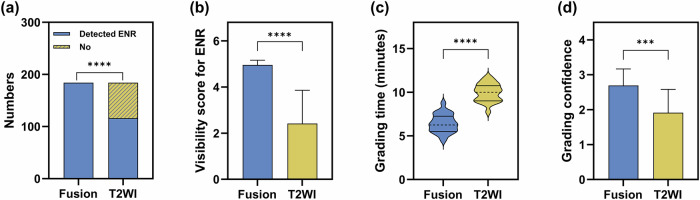
**a** Detection rate for ENR (χ^2^ test). **b** Visibility score for ENR (Mann–Whitney *U*-test). **c** Average time in grading CNFS (Independent samples *T*-test). **d** Confidence in grading CNFS (Mann–Whitney *U*-test) (*p < 0.05, **p < 0.01, ***p < 0.001, ****p < 0.0001)

A total of 184 cervical neural foramina (*N*_CNF_ = 184) were evaluated. On 3D-T2-FFE/FRACTURE fusion, the detection rate for ENR was 100% (184/184), which was higher than that on T2WI (116/184, 63.04%) (Fig. 4a). The visibility score for ENR, assessed on a 5-point Likert scale (1–5), was 5.00 (0.00) on 3D-T2-FFE/FRACTURE fusion and 2.00 (3.00) on T2WI. The visibility score for ENR on 3D-T2-FFE/FRACTURE fusion was higher than that on T2WI (Fig. 4b).

### Grade of CNFS

The average time taken for grading CNFS using the 3D-T2-FFE/FRACTURE fusion images was shorter, and the average confidence score was higher, compared to T2WI (Table 2 and Fig. 4).

**Fig. 5:**
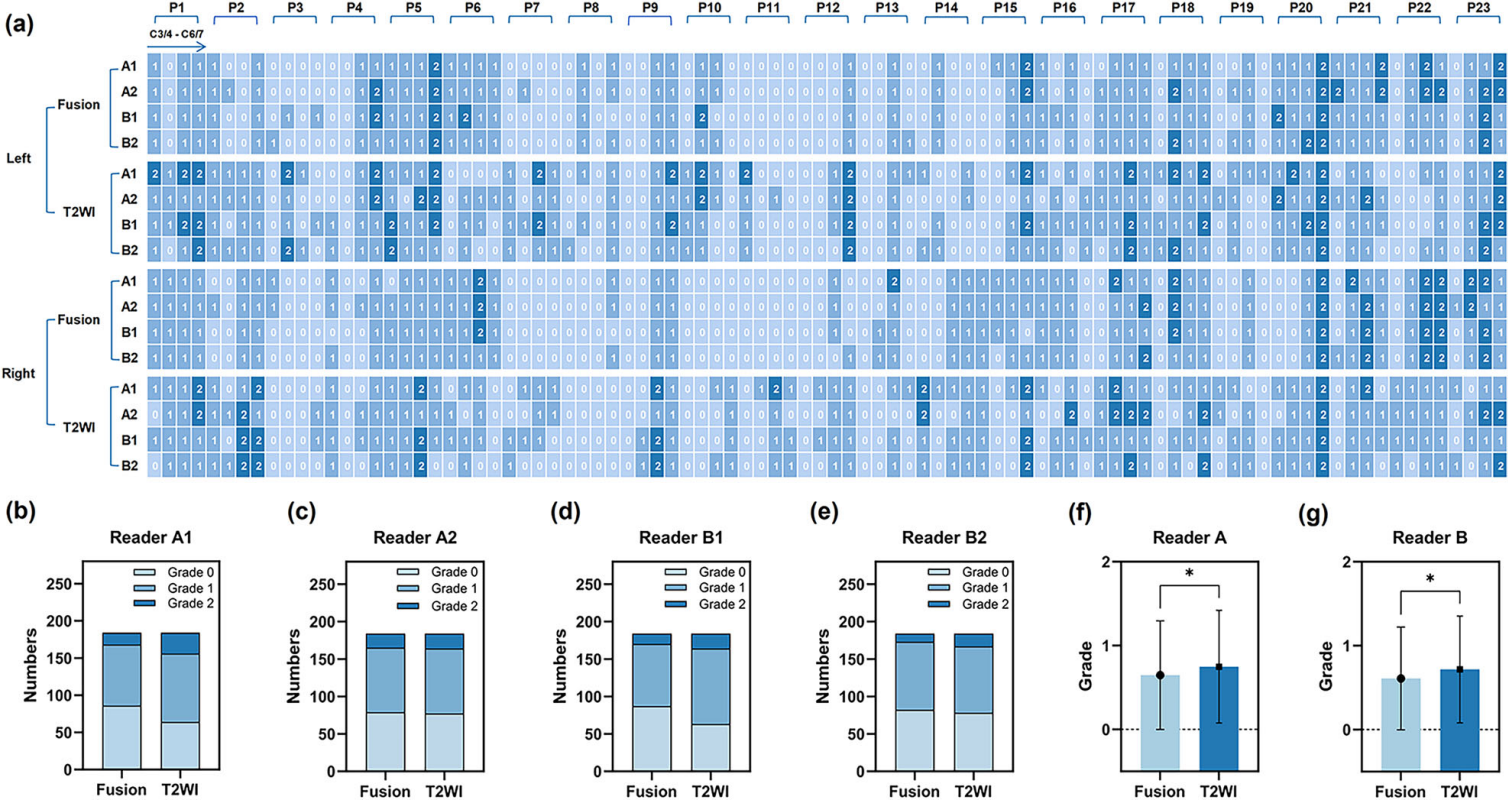
**a** CNFS grade distribution in 23 patients (P1–P23). **b**–**e** Number of each CNFS grade assessed by readers A and B. **f**, **g** Difference in CNFS grade between 3D-T2-FFE/FRACTURE fusion and T2WI (Mann–Whitney *U*-test). A1/2, reader A’s first/second read; B1/2, reader B’s first/second read (*p < 0.05)

The distribution of overall CNFS grade is shown in Fig. 5. Our findings demonstrated that the proportion of grade 1 and grade 2 on T2WI was higher than that on 3D-T2-FFE/FRACRURE fusion (Fig. 5a–e). In addition, the Mann–Whitney *U*-test showed that the overall CNFS grade on T2WI was slightly higher than that on 3D-T2-FFE/FRACTURE fusion (Fig. 5f, g).

The intra-reader agreement for CNFS grading was nearly perfect on 3D-T2-FFE/FRACTURE fusion images (*κ* = 0.791–0.837), and moderate on T2WI (*κ* = 0.436–0.636). The inter-reader agreement for CNFS grading was substantial on 3D-T2-FFE/FRACTURE fusion images (*κ* = 0.774–0.789), and moderate on T2WI (*κ* = 0.447–0.576) (Table 4).

**Table 4:** Reliability for the grade of CNFS on 3D-T2-FFE/FRACTURE fusion and T2WI

		Fusion		T2WI	
		*κ*	*p*	*κ*	*p*
Intra-reader agreement	A1 vs A2	0.837	< 0.001	0.436	< 0.001
		[0.771–0.903]		[0.332–0.539]	
	B1 vs B2	0.791	< 0.001	0.636	< 0.001
		[0.714–0.867]		[0.540–0.732]	
Inter-reader agreement	A1 vs B1	0.774	< 0.001	0.576	< 0.001
		[0.698–0.849]		[0.474–0.678]	
	A2 vs B2	0.789	< 0.001	0.447	< 0.001
		[0.714–0.863]		[0.342–0.553]	

All data were given as a weighted Cohen Kappa coefficient (*κ*) with a two-sided 95% CI

*CNFS* cervical neural foraminal stenosis, *Fusion* 3D-T2-FFE/FRACTURE fusion, *T2WI* T2-weighted image, *A1* first reading of reader A, *A2* second reading of reader A, *B1* first reading of reader B, *B2* second reading of reader B

### Clinical correlation between the grade of CNFS and NDI/NPS

After correcting for age, sex, BMI, and months of disease duration, CNFS grades based on 3D-T2-FFE/FRACTURE fusion were moderately associated with NDI scores (ρ = 0.49, *p* < 0.05) and NPS scores (ρ = 0.55, *p* < 0.05). However, CNFS grades based on T2WI were not significantly associated with NDI (ρ = 0.33, *p* > 0.05) or NPS (ρ = 0.25, *p* > 0.05).

## Discussion

Severe CNFS may compress cervical nerve roots, leading to cervical radiculopathy. The correct diagnosis and grading of CNFS are important for planning surgery. In this study, we proposed a novel method based on 3-T MR nerve/bone fusion to evaluate cervical neural foramina and cervical nerve roots. Compared with T2WI, the MR nerve/bone fusion imaging provides reliable and reproducible images with higher inter- and intra-reader agreement, which can clearly depict cervical nerve root and bony structures of cervical neural foramina together. The severity of CNFS based on MR nerve/bone fusion imaging is moderately associated with clinical symptoms.

First of all, we assessed the reliability and reproducibility of 3D-T2-FFE/FRACTURE fusion qualitatively and quantitatively. The overall image quality of 3D-T2-FFE/FRACTURE fusion images was excellent. We have minimized the artifacts at the lung apex as much as possible, but a few still remain. These artifacts may be attributed to the respiratory motion or magnetic field inhomogeneity. However, these artifacts did not interfere with our observation of the cervical neuroforamina. And our results on healthy participants showed that 3D-T2-FFE/FRACTURE fusion images were reliable and reproducible.

Then, 3D-T2-FFE/FRACTURE fusion images were used on patients with suspected cervical radiculopathy and compared with T2WI. The ENR is sometimes not visible on axial T2WI, leading to a relatively low reliability in grading CNFS. In this study, the detection rate for ENR on 3D-T2-FFE/FRACTURE fusion images was higher than that on T2WI. In addition, the visibility score for ENR on 3D-T2-FFE/FRACTURE fusion images was also higher than that on T2WI. Therefore, the visibility of ENR on 3D-T2-FFE/FRACTURE fusion was significantly better than that on T2WI. This may help in grading CNFS based on Kim’s grading system. When ENR is not visible, the width of the contralateral ENR at the level of the superior articular process or the width between the posterior margin of the vertebral artery and the anterior margin of the superior articular process was alternatively used [10]. In the process of image assessment, we found that this method may overestimate the size of ENR. This may be the reason why the overall CNFS grade on T2WI was slightly higher than that on 3D-T2-FFE/FRACRURE fusion.

Our results showed that the intra-reader and inter-reader agreements for CNFS grading on 3D-T2-FFE/FRACTURE fusion were significantly higher than those on T2WI. A previous study [10] evaluating the reliability of Kim’s grading system based on axial T2WI obtained moderate inter-reader agreement, which was similar to our results on T2WI. This means that the grading system based on 3D-T2-FFE/FRACTURE fusion is easier and more reliable than T2WI. This may be attributed to the better visibility of ENR and cervical neural foramen on 3D-T2-FFE/FRACTURE fusion.

The total scanning time for 3D-T2-FFE and FRACTURE sequences is approximately 8 min. The fusion of 3D-T2-FFE and FRACTURE sequences was performed on the Philips MR workstation (IntelliSpace Portal, ISP) and took less than 2 min. Although this total examination time is longer than that of T2WI, it remains clinically acceptable. Furthermore, our results showed that CNFS grading can be completed faster using 3D-T2-FFE/FRACTURE fusion images than T2WI. This is because these fusion images depict the cervical neuroforamina and ENR more clearly. Additionally, readers felt more confident when grading with 3D-T2-FFE/FRACTURE fusion images. However, broader clinical adoption may be limited by the fact that the acquisition and post-processing of 3D-T2-FFE and FRACTURE sequences are available on Philips platforms in this study. Although other manufacturers also offer nerve and bone imaging sequences, such as double-echo steady-state (DESS) and ZTE sequences, the feasibility of fusing these sequences from different manufacturers for the CNFS grading remains an area that requires further exploration.

Finally, an analysis of the clinical correlation between the grade of CNFS and NDI/NPS was conducted. Our results showed that a moderate correlation was obtained based on 3D-T2-FFE/FRACTURE fusion after correcting for age, sex, BMI, and months of disease duration. However, no clinical correlation was found when the grade of CNFS was evaluated on T2WI. To the best of our knowledge, there was no study analyzing the correlation between Kim’s grade of CNFS and NDI/NPS. A previous study [8] demonstrated a negligible correlation between the grade of CNFS (Park’s grading system [30]) and NDI/visual analog scale. As mentioned above, the overall CNFS grade on T2WI was slightly higher than that on 3D-T2-FFE/FRACRURE fusion. This may be the reason why no clinical correlation was found when evaluating the grade of CNFS on T2WI. While our findings demonstrate a moderate correlation between CNFS grades based on 3D-T2-FFE/FRACTURE fusion images and NDI/NPS, it is important to note that this correlation is statistically significant, but not very strong clinically.

Several limitations in this study need to be mentioned. Firstly, the sample size of patients in our study was relatively small. Although the correlation analysis yielded statistically significant results, the generalizability to a larger cohort may be limited. Therefore, it is essential to conduct further prospective studies with larger cohorts to enhance the clinical correlation and generalizability of the findings. Secondly, this was a single-center study, which may limit the applicability of the evaluations across different institutions. Thirdly, although CNFS grading on 3D-T2-FFE/FRACTURE fusion showed better correlations with patient symptoms (NDI/NPS), we could not definitively confirm its anatomical accuracy due to the lack of surgical validation. While symptom-based correlations may support clinical relevance, direct comparison to intraoperative findings remains essential to establish whether CNFS grades on 3D-T2-FFE/FRACTURE fusion truly reflect the degree of nerve root compression. Further studies focusing on surgical validation are critically needed to establish the clinical utility of the 3D-T2-FFE/FRACTURE fusion technique. Finally, the 3D-T2-FFE/FRACTURE fusion images used for evaluation were reconstructed in an axial orientation, using slice thickness and intervals equivalent to those of conventional axial T2WI images. Despite this standardization, aligning all image types to the exact same slice during analysis remained challenging, leading to some discrepancies in evaluating CNFS.

## Conclusion

Overall, 3D-T2-FFE/FRACTURE fusion imaging is more reliable in evaluating and grading CNFS compared with T2WI. The MR grade of CNFS based on 3D-T2-FFE/FRACTURE fusion imaging is associated with clinical symptoms. Therefore, the use of 3D-T2-FFE/FRACTURE fusion imaging in clinical practice may facilitate a one-stop shop and radiation-free approach to comprehensively evaluate CNFS.

## Supplementary information

ELECTRONIC SUPPLEMENTARY MATERIAL

## Data Availability

The datasets generated and analyzed during the current study are not publicly available.

## Abbreviations

3D-T2-FFE: 3D-T2-weighted fast field echo
CNFS: Cervical neural foraminal stenosis
ENR: Extraforaminal nerve root
FRACTURE: Fast field echo resembling a CT using restricted echo-spacing
ICC: Intraclass correlation coefficient
NDI: Neck disability index
NPS: Numerical pain scale
T2WI: T2-weighted images
WCNF: Width of the cervical neural foramen
WENR: Width of the extraforaminal nerve root
