# Supplementary material for: Grading cervical neural foraminal stenosis via 3-T MR nerve/bone fusion imaging compared with T2-weighted imaging
Source: Insights Imaging. 2025 Oct 16;16:218. doi: 10.1186/s13244-025-02094-3 (PMC12532963; doi:10.1186/s13244-025-02094-3)
Supplement: Supplementary file 1 — ELECTRONIC SUPPLEMENTARY MATERIAL [file 13244_2025_2094_MOESM1_ESM.pdf]

Grading cervical neural foraminal stenosis via 3T MR nerve/bone fusion imaging compared with T2-weighted imaging

ELECTRONIC SUPPLEMENTARY MATERIAL

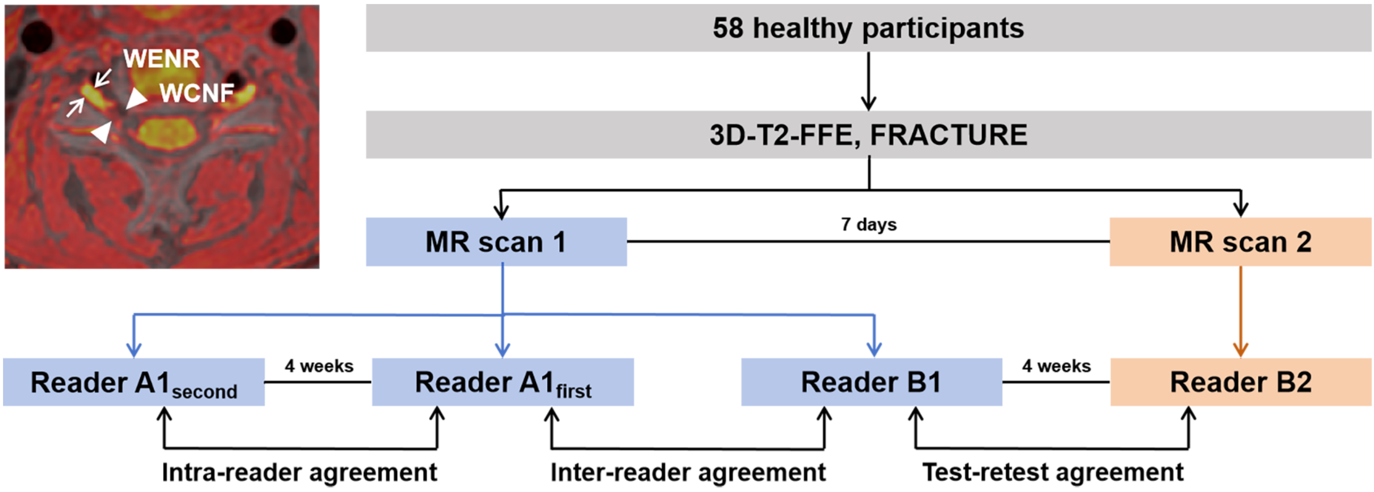

Fig. S1 Example of 3D-T2-FFE/FRACTURE fusion image and flowchart of the assessment for the reliability and reproducibility of the 3D-T2-FFE/FRACTURE fusion technique. WCNF, width of the cervical neural foramen; WENR, width of the extraforaminal nerve root; Reader $A1_{first}$, first reading of reader A about MR scan 1; Reader $A1_{second}$, second reading of reader A about MR scan 1; Reader B1, reading of reader B about MR scan 1; Reader B2, reading of reader B about MR scan 2.

Table S1 Parameters for cervical conventional MRI sequences.

Parameters	3D-T2-FFE	FRACTURE	T1WI	T2WI	T2WI
**Slice orientation**	coronal	coronal	sagittal	sagittal	transverse
**Acquisition resolution (mm^3)**	1.2×1.2×1.2	1.2×1.2×1.2	0.7×0.88×3	0.9×0.9×3	0.6×0.8×3
**FOV (mm^2)**	180×180	180×180	160×180	160×180	160×160
**Acquisition time (min: s)**	04: 43	03: 33	01:58	01:53	02:10
**Number of signal averaged**	2	2	1.6	1	1.8
**TR (ms)**	11	20	474	2500	2500
**$TE_{(first)}$ (ms)**	-	2.3	-	-	-
**TE (ms)**	5.4	-	6.8	140	110
**Echo space (ms)**	-	2.3	-	-	-
**Number of echos**	1	6	1	1	1
**Flip angle (degree)**	30	15	80	90	90

FOV, field of view; TE, echo time; $TE_{(first)}$, the first TE; TR, repetition time.

Appendix S1

Neck Disability Index (NDI)

Instructions

This questionnaire has been designed to give your health practitioner information as to how your neck pain has affected your ability to manage in everyday life. Please answer every section and mark in each section only the ONE box which applies to you. We realise you may consider that two of the statements in any one section relate to you, but please just mark the box which most closely describes your problem.

Section 1: Pain intensity

☐ I have no pain at the moment.

☐ The pain is very mild at the moment.

☐ The pain is moderate at the moment.

☐ The pain is fairly severe at the moment.

☐ The pain is very severe at the moment.

☐ The pain is the worst imaginable at the moment.

Section 2: Personal care (washing, dressing)

☐ I can look after myself normally without causing extra pain.

☐ I can look after myself normally but it causes extra pain.

☐ It is painful to look after myself and I am slow and careful.

☐ I need some help but manage most of my personal care.

☐ I need help every day in most aspects of self-care.

☐ I do not get dressed, I wash with difficulty and stay in bed.

Section 3: Lifting

☐ I can lift heavyweights without extra pain.

☐ I can lift heavyweights but it gives extra pain.

☐ Pain prevents me from lifting heavyweights off the floor, but I can manage if they are conveniently positioned, for example on a table.

☐ Pain prevents me from lifting heavyweights, but I can manage light to medium weights if they are conveniently positioned.

☐ I can lift very lightweights.

☐ I cannot lift or carry anything at all.

Section 4: Reading

☐ I can read as much as I want to with no pain in my neck.

☐ I can read as much as I want to with slight pain in my neck.

☐ I can read as much as I want with moderate pain in my neck.

☐ I cannot read as much as I want because of moderate pain in my neck.

☐ I can hardly read at all because of severe pain in my neck.

☐ I cannot read at all.

Section 5: Headaches

☐ I have no headaches at all.

☐ I have slight headaches which come infrequently.

☐ I have moderate headaches which come infrequently.

☐ I have moderate headaches which come frequently.

☐ I have severe headaches which come frequently

☐ I have headaches almost all the time.

Section 6: Concentration

☐ I can concentrate fully when I want to without difficulty.

☐ I can concentrate fully when I want to with slight difficulty.

☐ I have a fair degree of difficulty in concentrating when I want to.

☐ I have a lot of difficulty in concentrating when I want to.

☐ I have a great deal of difficulty in concentrating when I want to.

☐ I cannot concentrate at all.

Section 7: Work

☐ I can do as much work as I want to.

☐ I can only do my usual work, but no more.

☐ I can do most of my usual work, but no more.

☐ I cannot do my usual work.

☐ I can hardly do any work at all.

☐ I cannot do any work at all.

Section 8: Driving

☐ I can drive my car without any neck pain.

☐ I can drive my car as long as I want with slight pain in my neck.

☐ I can drive my car as long as I want with moderate pain in my neck.

☐ I cannot drive my car as long as I want because of moderate pain in my neck.

☐ I can hardly drive at all because of severe pain in my neck.

☐ I cannot drive my car at all.

Section 9: Sleeping

☐ I have no trouble sleeping.

☐ My sleep is slightly disturbed (less than 1 hr sleepless).

☐ My sleep is mildly disturbed (1-2 hrs sleepless).

☐ My sleep is moderately disturbed (2-3 hrs sleepless).

☐ My sleep is greatly disturbed (3-5 hrs sleepless).

☐ My sleep is completely disturbed (5-7 hrs sleepless).

Section 10: Recreation

☐ I am able to engage in all my recreation activities with no neck pain at all.

☐ I am able to engage in all my recreation activities, with some pain in my neck.

☐ I am able to engage in most, but not all of my usual recreation activities because of pain in my neck.

☐ I am able to engage in a few of my usual recreation activities because of pain in my neck.

☐ I can hardly do any recreation activities because of pain in my neck.

☐ I cannot do any recreation activities at all.

References

[1] Vernon H, Mior S. The Neck Disability Index: a study of reliability and validity. *J Manipulative Physiol Ther* 1991 Sep;14(7):409-15.

[2] TRACsa Trauma Injury and Recovery. Clinical guidelines for best practice management of acute and chronic whiplash-associated disorders. Canberra: National Health and Medical Research Council; 2008.

Appendix S2

Numeric Pain Scale (NPS)

The numeric pain scale (NPS) uses whole numbers (**Fig. S2**). This format uses a discontinuous and segmented scale. The most common styles include a horizontal bar or line format. The line or bar is marked with whole numbers from 0 to 10. The patient is asked to mark on the scale to rate their pain and then the number is recorded. The length of the line is not essential for this scale.

**0**	**1**	**2**	**3**	**4**	**5**	**6**	**7**	**8**	**9**	**10**

No pain **Most pain**

Fig. S2 Numeric Pain Scale

References

[1] Breivik EK, Bjornsson GA, Skovlund E. A comparison of pain rating scales by sampling from clinical trial data. Clin J Pain 2000;16:22–8.

[2] Lundeberg T, Lund I, Dahlin L, Borg E, Gustafsson C, Sandin L, et al. Reliability and responsiveness of three different pain assessments. J Rehabil Med 2001;33:279–83.

[3] Mader TJ, Blank FS, Smithline HA, Wolfe JM. How reliable are pain scores?A pilot study of 20 healthy volunteers. J Emerg Nurs 2003; 29:322–5.
